# Global minds and local contexts: grand challenges and emerging opportunities in cultural psychology

**DOI:** 10.3389/fpsyg.2026.1768471

**Published:** 2026-02-05

**Authors:** Fanli Jia

**Affiliations:** Department of Psychology, Seton Hall University, South Orange, NJ, United States

**Keywords:** cultural psychology, cultural responsiveness, decolonization, global psychology, indigenous methodologies, WEIRD

During a conference in 2008, a senior researcher dismissed my thesis on culture and moral development: “There is no cultural difference; you don't have a good instrument.” This critique, which questioned not only my methodology but the very premise that culture matters in psychology, has stayed with me. Over the years, I have continued to encounter similar skepticism where cultural differences are minimized as “small mean differences” or dismissed as the result of “*p*-value hacking.” These moments reflect more than isolated academic disagreements; they reveal a tension at the heart of cultural psychology: the enduring challenge of reconciling the pursuit of psychological universals with the undeniable presence of cultural variability.

What began as a personal academic struggle soon revealed itself as part of a larger, systemic issue within the discipline. While psychology aspires to explain human behavior broadly, it has long been shaped by a narrow view of humanity, one dominated by a WEIRD (Western, Educated, Industrialized, Rich, and Democratic) perspective (Henrich et al., 2010). This overreliance introduces systematic biases that compromise the field's ability to produce findings that are inclusive and generalizable. Addressing this problem requires more than technical fixes; it demands a fundamental rethinking of the assumptions, methods, and frameworks that define psychological science. If we are to build a truly global discipline, we must move beyond simply acknowledging cultural differences and instead treat culture as central to understanding the mind (Wang, 2016). Cultural psychology offers a powerful framework for this transformation. Drawing on diverse cultural perspectives, the field has the potential to shift from token inclusion to the generation of knowledge that is deeply contextual, globally relevant, and open to multiple ways of understanding.

## The American-centric and WEIRD psychology

Psychology's claims to universal applicability have long been undermined by a persistent and well-documented sampling bias. In his landmark critique, Arnett (2008) revealed that 68% of research samples in psychology's most prominent journals came from the United States. More than a decade later, Thalmayer et al. (2021) revisited the same six journals and found that American dominance remained largely unchanged at 67%. Despite this stagnation, participation from other English-speaking and Western European countries increased, further narrowing the global diversity of the field's evidence base.

The consequence of this sampling bias extends beyond a gap in representation; it actively distorts and limits our foundational scientific inquiries. An example is the ongoing debate over the degree to which culture shapes basic cognitive and perceptual processes. Consider the domain of visual attention. Classic work suggests individuals from East Asian cultures (e.g., Chinese) tend to show a more holistic cognitive style, attending broadly to contextual fields, while Westerners (e.g., Americans) are more likely to display an analytic style, focusing on focal objects (Nisbett et al., 2001). This finding challenged the premise of universal cognitive processes. However, the hallmark of a maturing science is not the absence of disagreement, but the presence of a continuous, critical examination of evidence. This is exemplified by the critical reevaluation of the Müller-Lyer illusion, a classic “case study” of cultural perception. Amir and Firestone (2025) challenge the long-held narrative that susceptibility to this illusion is culturally determined, arguing instead for more universal perceptual mechanics and noting inconsistencies in the original cross-cultural evidence.

These cultural differences are not confined to perception; they manifest across multiple levels of psychological functioning from basic cognition to the sense of self. For example, Wang's (2004) work on autobiographical memory shows how independent self-construals promote detailed “me” memories, whereas interdependent self-construals foster schematic “we” memories. Remarkably, these culturally patterned cognitive styles are now detectable even in non-human systems 20 years later. Lu et al. (2025) found that large language models like GPT show a more holistic and interdependent orientation when prompted in Chinese vs. English, demonstrating that cultural signatures in human thought are being reproduced in the AI trained on our cultural outputs.

This is not a crisis for cultural psychology; it is the engine. These discussions bring us back to the table to ask better questions. It shifts the research focus from *whether* culture matters to *how, when, and why* it matters. Does culture create entirely different perceptual architectures, or does it tune with universal cognitive processes? The contrast between Nisbett's evidence for field-dependent attention and Amir & Firestone's challenge to the Müller-Lyer cases illustrates that the answer is not uniform across all cognitive domains. Likewise, evidence that large language models reproduce cultural cognitive styles demonstrates the robustness and replicability of these cultural patterns. This continuous, evidence-based discussion is precisely what is needed to build a non-WEIRD psychology. It guards against both the bias of assuming universality and the risk of essentializing difference. It forces the field toward greater methodological rigor, more precise theories, and a deeper investigation of the mechanisms.

The limitations of psychological science go beyond American-centric and WEIRD samples. Dudgeon and Bray (2024) highlight how Western-dominated frameworks often marginalize Indigenous understandings of healing, resilience, and wellbeing through epistemic injustice and the privileging of Western knowledge systems. Schulz et al. (2019) demonstrate that the individualistic and nonconforming traits common in WEIRD societies are not universal, but shaped by historical shifts. Building on this, Henrich (2020) argues that human cognition itself is a product of cultural evolution, shaped by the social, historical, and ecological contexts in which people live.

Cultural psychology invites us to see what mainstream psychology has often missed in three critical ways. First, it provides a theoretical foundation that foregrounds cultural evolution as central to understanding psychological diversity (Henrich, 2020). Rather than assuming universality, cultural psychology seeks to explain when and why psychological processes differ across societies. Second, it emphasizes the importance of indigenous knowledge systems and local meaning-making, contributing to efforts to decolonize psychological science (Smith, 2021). Recent work by Fish and Gone (2024) suggests that even decolonial approaches must evolve beyond simple critique to embrace anticolonial methodologies that center Indigenous self-determined futures such as agency, sovereignty, and forward vision. Third, it advocates for methodological transformation in psychological science. Krys et al. (2025) argue that advancing cultural psychology requires expanding research beyond currently overrepresented regions (e.g., WEIRD or Confucian societies) by including more diverse world cultures and adopting larger, more inclusive cross-cultural study designs. Achieving truly global psychology means integrating cultural context into every stage of the research process, from theory building to methodology to interpretation (Wang, 2006, 2016). This transformation extends to clinical and applied settings, where Western-based interventions may not translate across cultural contexts, requiring a fundamental rethinking of how we understand and support psychological wellbeing across diverse cultural settings (Turpin and Coleman, 2010).

## Global transformation: the rise of crowdsourced international collaborations

As psychology continues to count with its American and WEIRD-centric foundations, a new paradigm is gaining momentum, one that embraces global collaboration, and cultural accountability. This shift is powerfully embodied in the rise of crowdsourced international collaborations, where researchers across continents co-design studies, collect data, and interpret findings through multifaceted cultural lenses. Unlike a traditional model where research is designed and conducted within a single lab or a limited network, these collaborations are characterized by distributed leadership, collective decision-making, and a genuine valuing of diverse cultural insights and methodological contributions (Azevedo et al., 2019) (e.g., Introducing a Framework for Open and Reproducible Research Training).

The impact of this crowdsourced approach is already evident in a growing body of groundbreaking research. For example, Vlasceanu et al. (2024) and Doell et al. (2024) coordinated studies across 63 countries to investigate culturally informed climate interventions. Żemojtel-Piotrowska et al. (2024) examined collective narcissism in 61 countries, providing new insights into the cultural variability of group identity. The COVID-19 pandemic further catalyzed global collaboration, as evidenced by Brzósk et al.'s (2023) exploration of how cultural norms shaped health beliefs across 50 countries and Sawicki's et al. (2022) validation of the Fear of COVID-19 scale in 48 countries. These large-scale efforts are mirrored by smaller crowdsourced projects that intentionally involve student researchers, reflecting the mission of initiatives like Psi Chi's Network for International Collaborative Exchange (e.g., Cook et al., 2023; Killikelly et al., 2023; Knutson et al., 2023; Moussa Rogers et al., 2024; Szkody et al., 2024). This model even extends to data re-analysis initiatives, where researchers collectively re-analyze existing datasets to evaluate replicability and robustness (e.g., Aczel et al., in press).

To ensure these collaborations are not symbolic, attention must be given not only to participant representation but to equitable authorship (Coles et al., 2023) and substantive involvement in research design and theory-building from non-WEIRD cultural perspectives (e.g., Ghai et al., 2025). Without such inclusion, collaborations risk reinforcing research colonialism where non-Western contexts are reduced to data-collection sites that primarily serve and validate Western theoretical frameworks (Jia, in press). This imbalance is often obscured when collaboration processes lack transparency regarding whose questions, analyses, and interpretations ultimately drive the research. Therefore, true inclusivity is achieved when scholars from diverse cultural contexts hold decisive authority in evaluating a study's relevance, theoretical fit, and methodological appropriateness within their own cultural frameworks.

## Teaching psychology globally: toward cultural responsiveness in the classroom

As the field of psychology undergoes a global transformation, the challenge is not only to reform research practices but also to rethink how we teach psychology, especially in ways that are culturally inclusive and globally relevant. Teaching, like research, has long been shaped by Western assumptions about what counts as authoritative knowledge and who is recognized as a “foundational” figure in the discipline.

At a recent conference of the National Institute on the Teaching of Psychology, I led a brief interactive demonstration by asking attendees to write down the names of the psychologists they most often teach in their Introduction to Psychology course. The responses poured in quickly with names that are familiar, widely cited, and central to mainstream textbooks. Yet almost all of them shared three striking characteristics: they were Western, White, and Male (“WWM”). The absence of the non-WWM figures was not due to a lack of scholarly impact or theoretical relevance, but rather to the narrow lens through which the field continues to define psychological authority. This exercise made clear how these exclusions are embedded in our teaching practices and how urgently we need to expand our curricula to reflect the full diversity of psychological thought. This simple exercise highlighted how the cultural limitations of research are mirrored in how psychology is taught. Students around the world are often introduced to the field through a curriculum that reflects a narrow set of cultural assumptions, perspectives, and voices. These silences shape not only what students learn, but how they come to understand what psychology is and who it is for.

To systematically address these limitations, the International Competences for Undergraduate Psychology (ICUP) model offers a global framework for transforming undergraduate education through a lens of cultural responsiveness and inclusion (Cranney et al., 2025; Nolan et al., 2025a,b, 2026a,b). Far from a simple checklist, the ICUP model represents psychological education as a truly global endeavor. Its core strength lies in its culturally inclusive design, which is grounded in the principle that foundational psychological competences must be valuable and applicable across all personal, professional, and community contexts, irrespective of a student's cultural background or career path (Cranney et al., 2025).

The model's commitment to inclusivity is further demonstrated by its explicit linkage of psychology education to the United Nations Sustainable Development Goals (SDGs), thereby positioning the discipline as essential for tackling global challenges like health equity and climate action (Nolan et al., 2025b). This outward focus cultivates global citizenship, ethical awareness, and, most critically, cultural humility, equipping students to critically engage with and look beyond dominant Western paradigms. The practical relevance of this culturally-attuned framework is evidenced by its adaptation in 15 national contexts, demonstrating its remarkable global utility (teaching case studies can be found here: https://osf.io/5hj7g/).

## Cultural psychology's new era: a vision for the future

*Frontiers in Psychology—Cultural Psychology* section is committed to rectifying a longstanding imbalance: for too long, cultural psychology has been treated as an afterthought, a “side dish” to the “main course” of Western psychological science. This marginalization has resulted in an incomplete understanding of human behavior, where Western perspectives are the default and other cultural viewpoints are secondary. Our section is pioneering a transformation to place culture at the very center of psychological inquiry. We recognize that meaningful change requires more than symbolic representation; it demands a restructuring of how cultural psychology is conducted, evaluated, and disseminated.

This commitment drives our specific editorial priorities. We see particular promise in research that examines how individuals and communities navigate globalizing forces while maintaining cultural continuity, revealing the complex ways cultural-ecological systems shape human development, cognition, and wellbeing in ways that often diverge from established Western models. This commitment to exploring processes beyond traditional East-West dichotomies (e.g., Kitayama and Salvador, 2024) is complemented by a focus on multiculturalism and migration, which offers critical insights into how people negotiate cultural boundaries, manage value conflicts, and construct hybrid identities in an increasingly interconnected world. To ensure such research is transformative, we also embrace the growing momentum to decolonize psychological science, which goes beyond mere representation to fundamentally challenge the intellectual dependencies embedded in traditional frameworks and elevate Global Minority perspectives. Ultimately, this vision extends beyond advancing research to transforming how psychological knowledge is taught; we are committed to developing a curriculum and research that reflects a truly global understanding of psychology, preparing the next generation of psychologists to approach the field with cultural humility and sensitivity.

Realizing this vision and supporting the groundbreaking research entails embracing alternative theories and methodologies, which is an institutional challenge as much as an intellectual one. Funding agencies and universities must lead this shift by creating equitable grant streams and reforming tenure criteria to actively support the community-engaged research, Indigenous scholarship, and non-WEIRD frameworks that generate these alternatives. Journals and editors are essential partners in this work. They can mandate “Context Justification” sections, requiring authors to detail the cultural parameters and limitations of their findings regardless of a study's sample origin (including both Global Majority and Global Minority contexts). Concurrently, diversifying reviewer pools to include regional and cultural experts creates the necessary editorial space for these alternative approaches to be rigorously validated. Within academic departments, this means moving beyond token inclusion to making strategic hires of scholars whose work inherently challenges the mainstream, ensuring these perspectives reshape core theory. Finally, transforming pedagogy through frameworks like the ICUP is how these alternative understandings become the new foundation for the next generation.

Looking ahead, our section is committed to being an active catalyst in the reshaping of psychological science. Our editorial team brings together scholars from diverse backgrounds dedicated to amplifying voices from non-Western contexts and implementing initiatives that support research grounded in global knowledge systems and culturally embedded experiences. Moving beyond surface-level comparisons, we advocate deep, context-sensitive approaches that illuminate how cultural meaning systems shape the mind. This coordinated, multi-level commitment is our tangible pathway from critiquing a WEIRD-centric past to constructing a genuinely equitable and globally relevant science of human behavior.
